# Supplementary material for: T-cell Redirecting Therapies for the Treatment of B-cell Lymphomas: Recent Advances
Source: Cancers (Basel). 2021 Aug 25;13(17):4274. doi: 10.3390/cancers13174274 (PMC8428367; doi:10.3390/cancers13174274)
Supplement: Supplementary file 1 [file cancers-13-04274-s001.zip › cancers-1338145-supplementary.pdf]

cancers

MDPI

Table S1. CD19 CAR T-cell Trials in Mantle Cell Lymphoma.

Characteristics	ZUMA-2	TRANSCEND NHL-001
Reference	Wang et al. [1]	Palomba et al. [2]
Product	Brexu-cel	Liso-cel
Patients infused	68	32
Median age (range)	65 (38–79)	67 (36–80)
Median follow-up (months)	17.5	5.9
Bridge, %	37	53
Flu/Cy	500/30 × 3 days	300/30 × 3 days
Dose of CAR-T	2×10^6/kg	50–100 × 10^6
ORR, %	92	84
CR, %	67	66
PFS, %	59% @ 15 months	-
OS, %	76% @ 15 months	-
All grade CRS, %	91	50
CRS grade ≥3, %	15	3
All grade ICANS, %	63	34
ICANS grade ≥3 %	31	12

CR, complete response; CRS, cytokine release syndrome; Cy, cyclophosphamide; Flu, fludarabine; ICANS, immune effector cell-associated neurotoxicity; ORR, overall response rate; OS, overall survival; PFS, progression-free survival.

Table S2. CD19 CAR T-cell Trials in Indolent/Follicular Lymphoma.

Characteristics	ZUMA-5	ELARA
Reference	Jacobson et al. [3]	Schuster et al. [4]
Product	Axi-cel	Tisa-cel
Patients infused	146 (FL: 124; MZL: 22)	97 FL
Median age (range)	61 (34–79)	57 (29–73)
Median follow-up (months)	17.5	11
Bridge	0%	44%
Flu/Cy	500/30 × 3 days	250/25 × 3 days (or Bendamustine 90 × 2 days)
Dose of CAR-T	2×10^6/kg	0.6–6 × 10^8 (median of 2.06 × 10^6/kg)
ORR, %	92	86.2
CR, %	76	66
PFS, %	73.7% @ 12 months	76% @ 6 months
OS, %	92.9% @ 12 months	Not reached
All grade CRS, %	82	48.5
CRS grade ≥3, %	7	0
All grade ICANS, %	60	9.3
ICANS grade ≥3, %	19	1

CR, complete response; CRS, cytokine release syndrome; Cy, cyclophosphamide; Flu, fludarabine; ICANS, immune effector cell-associated neurotoxicity; ORR, overall response rate; OS, overall survival; PFS, progression-free survival.

References

1. Wang, M.; Munoz, J.; Goy, A.; Locke, F.L.; Jacobson, C.A.; Hill, B.T.; Timmerman, J.M.; Holmes, H.; Jaglowski, S.; Flinn, I.W.; et al. KTE-X19 CAR T-cell therapy in relapsed or refractory mantle-cell lymphoma. *N. Engl. J. Med*. **2020**, *382*, 1331–1342; doi:10.1056/nejmoa1914347.
2. Palomba, M.L.; Gordon, L.I.; Siddiqi, T.; Abramson, J.S.; Kamdar, M.; Lunning, M.A.; Maloney, D.G.; Andreadis, C.; Arnason, J.E.; et al. Safety and Preliminary Efficacy in Patients with Relapsed/Refractory Mantle Cell Lymphoma Receiving Lisocabtagene

Maraleucel in Transcend NHL 001. 2020. Available online: https://ash.confex.com/ash/2020/webprogram/Paper136158.html (5 December 2020).
3. Locke, F.L.; Rossi, J.M.; Neelapu, S.S.; Jacobson, C.A.; Miklos, D.B.; Ghobadi, A.; Oluwole, O.O.; Reagan, P.M.; Lekakis, L.J.; Lin, Y.; et al. Tumor burden , in fl ammation , and product attributes determine outcomes of axicabtagene ciloleucel in large B-cell lymphoma. *Blood Adv.* **2020**, *4*, 4898–4911; doi:10.1182/bloodadvances.2020002394.
4. Schuster, S.J.; Dickinson, M.J.; Dreyling, M.H.; Martinez, J.; Kolstad, A.; Butler, J.P.; Ghosh, M.; Popplewell, L.; Chavez, J.C.; Bachy, E.; et al. Efficacy and safety of tisagenlecleucel (Tisa-cel) in adult patients (Pts) with relapsed/refractory follicular lymphoma (r/r FL): Primary analysis of the phase 2 Elara trial. *J. Clin. Oncol.* **2021**, *39*, 7508–7508; doi:10.1200/JCO.2021.39.15_suppl.7508.
